# Early diagnosis improving the outcome of an infant with epileptic encephalopathy with cytoplasmic FMRP interacting protein 2 mutation: Case report and literature review

**DOI:** 10.1097/MD.0000000000017749

**Published:** 2019-11-01

**Authors:** Min Zhong, Shuang Liao, Tingsong Li, Peng Wu, Yanqin Wang, Fangrui Wu, Xiujuan Li, Siqi Hong, Lisi Yan, Li Jiang

**Affiliations:** a Department of Neurology, Children's Hospital of Chongqing Medical University; b China International Science and Technology Cooperation Base of Child Development and Critical Disorders, Chongqing Engineering Research Center of Stem Cell Therapy, Ministry of Education Key Laboratory of Child Development and Disorders; c National Clinical Research Center for Child Health and Disorders; d Qianjiang Central Hospital of Chongqing, Chongqing, China.

**Keywords:** CYFIP2, epileptic encephalopathy, hypsarrhythmia, vigabatrin, whole-exome sequencing

## Abstract

**Rationale:**

Early infantile epileptic encephalopathy (EIEE) 65 was recently shown to be caused by the cytoplasmic FMRP interacting protein 2 (*CYFIP2*) mutation. To date, only 5 cases have been reported in two articles, and all the outcomes in all cases were poor.

**Patient concerns:**

In this study, we reported an 8-month-old girl with a 1 month-long history of seizures and developmental delay. Over 1 month later, she developed epileptic spasms in clusters with hypsarrhythmia on electroencephalography.

**Diagnosis:**

The patient was diagnosed with EIEE 65 and trio-based whole-exome sequencing revealed a causative de novo *CYFIP2* mutation c.260G >T (p.Arg87Leu).

**Interventions:**

The proband was successively treated with multiple antiepileptic drugs, including levetiracetam, phenobarbital, VitB6, topiramate, methylprednisolone, prednisone, valproic acid and vigabatrin.

**Outcomes:**

After resistance to multiple anti-epileptic drugs over 2 months of treatment, she finally achieved seizure-free several days after vigabatrin administration and her developmental delay steadily improved.

**Lessons: Our:**

case confirmed that *CYFIP2* was the pathogenic gene of EIEE 65. We also first demonstrated vigabatrin might be effective for control of seizures and helpful for the improved outcomes of these patients.

## Abstract 2

Supplemental Digital Content is available in the text

## 1 Introduction

Epileptic encephalopathy (EE) was formally defined as epileptic activity that contributes to severe and progressive cognitive and behavioral impairment above and beyond what might be expected from the underlying pathology alone. Most EE cases are affected by onset in the early infantile year of life (EIEE),^[1,2]^ and nearly all patients experience a devastating process with a high risk of comorbidities and poor prognosis. In addition to the cerebral structural abnormalities found in a minority of these diseases, accumulating evidence indicates a major genetic component in the etiology of EIEEs. In recent years, numerous causative genes (>80) have been identified in epilepsy patients^[3]^ providing greater insight into the pathogenesis of EIEEs. To date, about 70 single-gene mutations associated with EIEEs have been submitted to the Online Mendelian Inheritance in Man database (www.omim.org). Several months ago, 4 cases of EIEE were reported to have cytoplasmic FMRP interacting protein 2 (*CYFIP2*) gene mutations that were designated EIEE 65 (OMIM: 618008).^[4]^

Here, we present the case of a baby girl with global intellectual disability and refractory epilepsy. Whole-exome sequencing (WES) of the proband identified a de novo missense mutation of the *CYFIP2* gene. We also compared our findings with those of 5 previously reported cases.

## 2 Methods

### 2.1 Ethical approval

Ethical approval for this study was obtained from the Institutional Review Board, Children's Hospital of Chongqing Medical University (2018-64). Informed consent was obtained from the patient's parents.

### 2.2 Patient information

Here, we report the case of a baby girl born to non-consanguineous Chinese parents at 40 weeks and 2 days of gestation (G1P1). Her mother had a cold during the first 6 months of pregnancy. The girl was born by normal vaginal delivery with a birth weight of 3010 g, body length of 50.5 cm and head circumference of 33 cm. The child was unable to support her head and roll over until she was 5 months of age.

The child was admitted to a local hospital for rehabilitation because of developmental delay; however, generalized tonic-clonic seizures (GTCSs) were identified by the clinicians and levetiracetam (35 mg/kg.d) was administered. Phenobarbital was added but stopped 2 weeks later. The seizures did not subside over the following month and the patient was transferred to our neurologic ward at the age of 6 months and 8 days. On admission, she had 10 GTCSs/d (Video 1), was still unable to support her head and responded poorly to voices and light. No characteristic dysmorphic features were observed except for a small forehead. According to the World Health Organization Anthro guidelines, the patient's anthropometric *Z*-scores were as follows: body weight 7 kg (*Z*-score, −2.17 standard deviations [SD]), body length 65 cm (*Z*-score, −2.75 SD), and head circumference 45 cm (*Z*-score, 0.02 SD).

During hospitalization, we added topiramate (6.5 mg/kg.d) and vitamin B6 to the patient's treatment regimen, but her seizures deteriorated and 10 days after hospitalization she developed epileptic spasms (Video 2) in clusters with hypsarrhythmia on electroencephalography (EEG). The patient was diagnosed as West syndrome, and methylprednisolone (15 mg/kg.d × 3 days) was administered by intravenous injection followed by oral prednisone (20 mg/kg.d) and valproic acid (30 mg/kg.d), but without improvement. Seizures occurred over 30 times a day and gastric tube feeding was started because of feeding problems. Finally, vigabatrin (50 mg/kg.d) was started at the request of the parents After 2 to 3 days, the patient had no seizures and she was able to suck milk dispensing with gastric tube feeding. The patient was seizure-free and discharged 33 days after admission. During the 1-month follow-up, the seizures were totally controlled, and her psychomotor development improved (Fig. 1 A).

**Figure 1:**
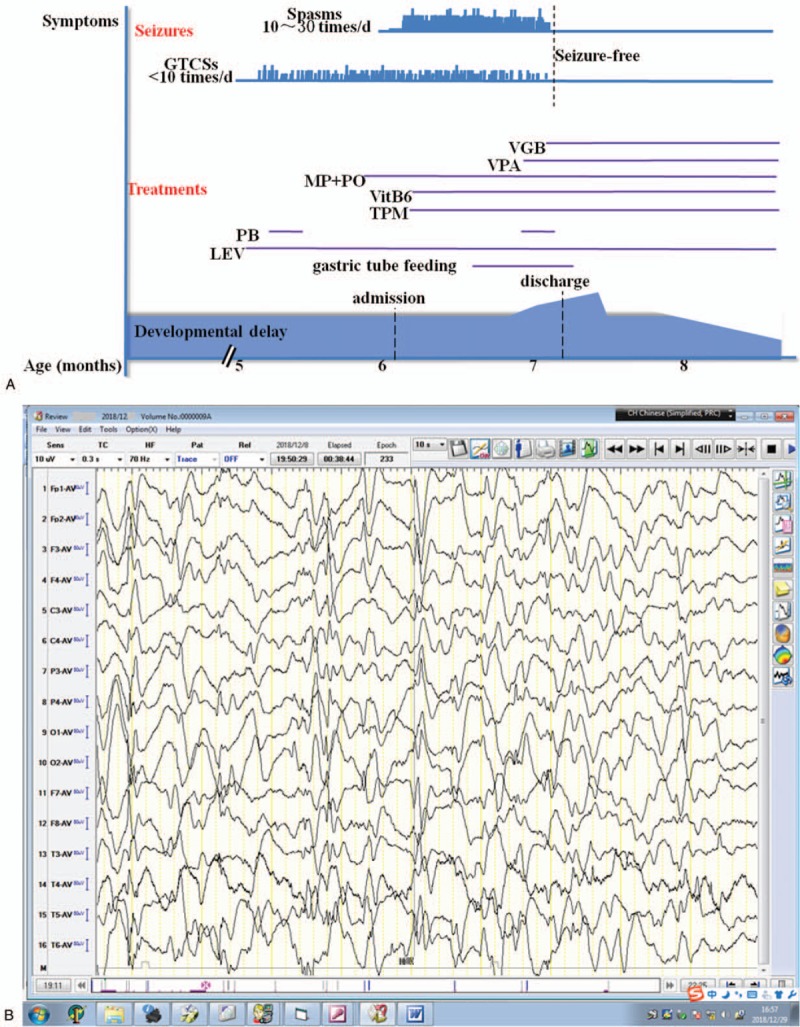
Summary of the clinical course of the patient (A). Seizures occurred approximately 10 times/d during the first month after the seizure onset. One and a half months later, the patient's condition deteriorated with epileptic spasms occurring in clusters accompanied by hypsarrhythmia on EEG (B). Several days after adding vigabatrin, the patient became seizure-free and her condition steadily improved, but still with hypsarrhythmia 1 mo later (C). EEG = electroencephalography.

**Figure 1 (Continued):**
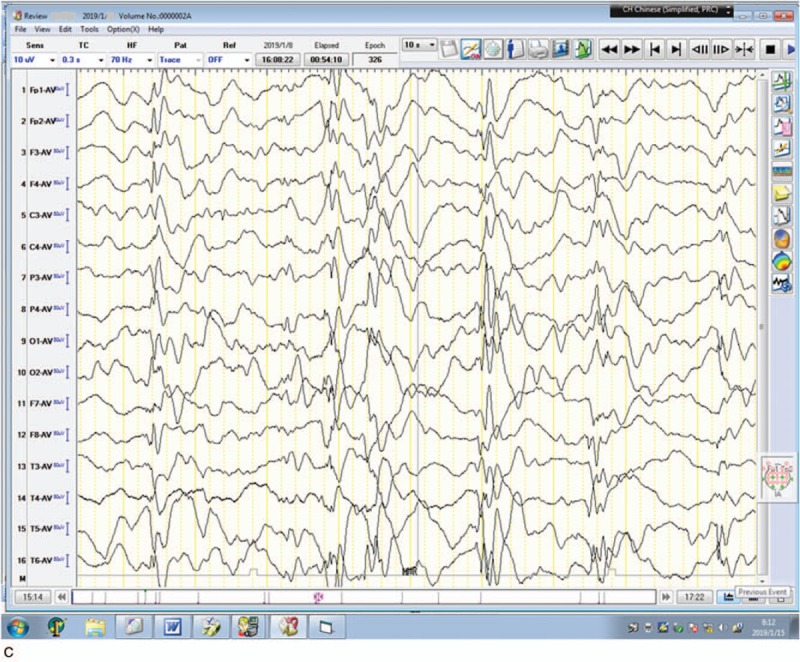
Summary of the clinical course of the patient (A). Seizures occurred approximately 10 times/d during the first month after the seizure onset. One and a half months later, the patient's condition deteriorated with epileptic spasms occurring in clusters accompanied by hypsarrhythmia on EEG (B). Several days after adding vigabatrin, the patient became seizure-free and her condition steadily improved, but still with hypsarrhythmia 1 mo later (C). EEG = electroencephalography.

The magnetic resonance imaging scans showed increased extra-axial spaces. Interictal EEG showed preliminary spike- and/or spark-slow waves across the whole brain, and this later developed to hypsarrhythmia (Fig. 1 B and C). Blood/urinary metabolic screening results were normal.

### 2.3 Molecular genetic analysis

Trio-based WES and copy number variation detection was performed on the proband and her parents by Chigene Biotech (Beijing, China).

**Figure 2:**
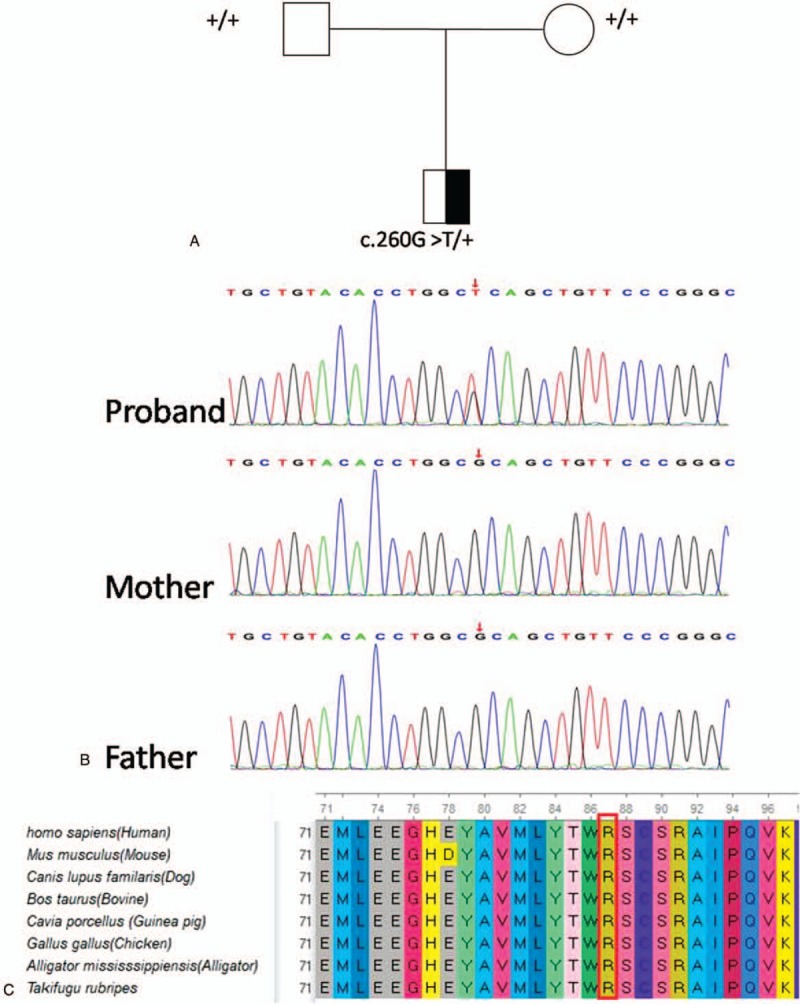
Whole-exome sequencing showed a de novo heterozygous mutation c.260G >T in the *CYFIP2* gene (A and B). The *CYFIP2* missense mutation (red arrow) is located in a highly conserved amino acid sequence among representative species (C). CYFIP2 = cytoplasmic FMRP interacting protein 2.

No pathogenic copy number variants were found. WES revealed a de novo heterozygous mutation c.260G>T, p.Arg87Leu (NM_001037333) in Exon 4 of the *CYFIP2* gene; this was confirmed by Sanger sequencing (Fig. 2A and B). Her parents were clinically healthy and did not carry this *CYFIP2* mutation. The child's diagnosis was revised as EIEE 65 at the age of 8 months.

The amino acid residue encoded by the *CYFIP2* gene was evolutionarily conserved among representative species predicted using the GERP++ (Fig. 2C), phyloP and phastCons tools. The missense mutation was predicted to be a deleterious variant by multiple in silico tools including Polyphen2_HDIV, Polyphen2_HVAR, MutationTaster, M-CAP and REVEL.

### 2.4 Literature review

Five cases of *CYFIP2* mutations were identified by searching the PubMed database (https://www.ncbi.nlm.nih.gov/pubmed/).^[4,5]^ All the patients were born to non-consanguineous parents and had no similar familial history. They developed early-onset epilepsy and global intellectual disability. Their clinical features are summarized in Table 1.

**Table 1:**
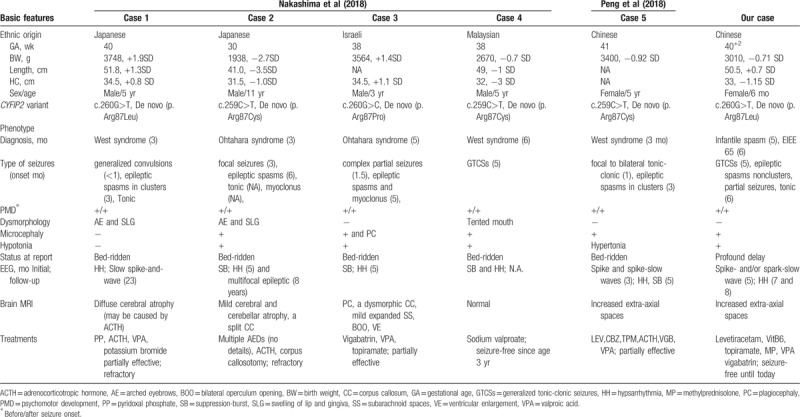
Clinical characteristics of confirmed patients with *CYFIP2* mutations from the literatures and our report.

## 3 Discussion

*CYFIP1* and *CYFIP2*, which are members of the highly conserved *CYFIP* gene family, are components of the WASP-family verprolin-homologous protein (WAVE) regulatory complex, which is involved in actin dynamics. WAVE family proteins play central roles in actin remodeling, axon elongation, dendritic spine morphogenesis, and synaptic plasticity.^[4,6]^

In a Drosophila model, mutants with only 1 CYFIP family homolog (dCYFIP) have shorter synapse terminals and a higher number of buds than their wild-type counterparts, which is indicative of impaired synapse growth. These abnormalities suggest that dCYFIP may regulate endocytosis and/or vesicle recycling by inhibiting F-actin assembly.^[6,7]^

In recent years, accumulating evidence suggests that CYFIP is closely associated with human cognition and/or behavior. Ten de novo *CYFIP2* variants (6 intronic, 1 synonymous, and 3 missense) have been reported in patients with autism and developmental disorders.^[8,9]^ Moreover, novel *CYFIP2* variants have recently been identified in 5 patients with severe early-onset epileptic encephalopathies by 2 different research groups^[4,5]^ and these pathogenic mutations have been functionally verified.^[4]^ These *CYFIP2* mutations were designated EIEE 65 (OMIM database). Interestingly, the only 2 heterozygous *CYFIP2* mutations found in these 5 cases affect the same amino acid residue (p.Arg87Cys, p.Arg87Pro, and p.Arg87Leu).^[4,5]^ Bioinformatics analysis and other research indicates that these *CYFIP2* variants cause gain-of-function of the WAVE signaling pathway, resulting in dysregulation of the neurological function in the brain. These mutations are thought to contribute to the pathogenesis of this form of EE, although further studies are needed to fully elucidate the biological functions of the wild-type and mutant *CYFIP2*. In addition to the *CYFIP2* mutation, the patient in Case 5 also had a de novo lysine methyltransferase 2D (*KMT2D*) mutation. Mutations in the *KMT2D* gene are reported mainly in association with Kabuki syndrome; thus, it is possible that the refractory epilepsy and developmental delay observed in this patient were associated with the *KMT2D* mutation.^[5]^

All 5 of the cases of early-onset EE reported with *CYFIP2* mutations were primarily diagnosed as Ohtahara syndrome (n = 2) or West syndrome (n = 3). Furthermore, all of the patients had profound developmental delay and refractory epilepsy, with recurrent GCTSs and/or focal seizures and epileptic spasms. Patients 1, 2, 3, and 5 had hypsarrhythmia and/or suppression-burst pattern on EEG, and responded poorly to multiple anti-epileptic drugs. Patient 4 had been seizure-free with valproic acid treatment for nearly 3 years and had only frequent generalized epileptiform activities on EEG during the whole course of the disease.

In our patient, psychomotor developmental delay was observed as early as 2 months of age. No improvement was observed after rehabilitation, and the delay was exacerbated after the onset of epilepsy. Aged 5 months, she developed GCTSs with spike- and/or spark-slow wave on EEG. The frequency of the seizures was not reduced by levetiracetam, topiramate, and vitamin B6 treatment and approximately 1 month later, her condition evolved to West syndrome. The patient responded poorly to treatment with corticosteroids and valproic acid. Fortunately, her seizures were almost completely controlled by vigabatrin, and her psychomotor response also improved. The patient did not have any seizures during the 1-month follow-up and developmental delay steadily improved, although hypsarrhythmia was still present on her EEG (Fig. 1 C). Here, we have reported the case of the earliest genetic diagnosis (8 months) of EIEE 65, with the patient responding well to treatment. The therapeutic effects and prognosis of early diagnosis and intervention in such cases will be clarified by long-term follow-up.

In conclusion, we have reported a case of EIEE 65 with a de novo heterozygous mutation of the *CYFIP2* gene, resulting in the (p.Arg87Leu) variant, which may be a pathogenic hot spot in *CYFIP2* associated with EIEE 65. Based on the case presented here and the previously reported cases, this newly identified form of EIEE appears to be refractory to multiple treatments. Thus, the results of this study expand the clinical spectrum of *CYFIP2* mutations and provide the first evidence indicating that vigabatrin is effective for controlling seizures and improving outcome in this patient population.

## Acknowledgments

We thank the patient and her family members who participated in this study. We also thank Prof. Fei Yin and Nan Pang (Xiangya Hospital, Central South University, Changsha, China) provided more details of the case 5.

## Author contributions

**Data curation:** Min Zhong, Yanqin Wang, Fangrui Wu, Lisi Yan.

**Formal analysis:** Min Zhong, Shuang Liao, Peng Wu.

**Funding acquisition:** Min Zhong.

**Investigation:** Min Zhong, Shuang Liao, Tingsong Li, Peng Wu, Yanqin Wang, Xiujuan Li.

**Methodology:** Min Zhong, Tingsong Li, Li Jiang.

**Supervision:** Min Zhong, Xiujuan Li, Siqi Hong, Li Jiang.

Min Zhong orcid: 0000-0002-0546-9663.

## Supplementary Material

Supplemental Digital Content

## Supplementary Material

Supplemental Digital Content
